# Mental‐Health Help‐Seeking Among Muslims in the Liverpool City Region: A System‐Informed Qualitative Study of Knowledge, Attitudes, Practices

**DOI:** 10.1111/hex.70663

**Published:** 2026-04-05

**Authors:** Ashraf Tannerah, Amy Webster, Shelley O'Connor, Charlie Douglas‐Brown, Imran Khan, Oladayo Bifarin

**Affiliations:** ^1^ School of Nursing and Advanced Practice, Faculty of Health Liverpool John Moores University Liverpool UK; ^2^ Community Service Alder Hey Children's NHS Foundation Trust Liverpool UK; ^3^ Centre for Primary Care, Wolfson Institute of Population Health, Faculty of Medicine and Dentistry Queen Mary University of London London UK; ^4^ Mental Health Research for Innovation Centre (M‐RIC) Mersey Care NHS Foundation Trust Liverpool UK

**Keywords:** community engagement, cultural safety, help‐seeking, mental health inequalities, muslim communities

## Abstract

**Background:**

Muslims in United Kingdom (UK) minority contexts remain underrepresented in mental health research, despite evidence of unequal access and experience. In deprived and superdiverse settings such as the Liverpool City Region (LCR), culturally unsafe encounters, low service literacy and stigma may combine to delay help‐seeking and widen inequities.

**Aim:**

To explore knowledge, attitudes and practices (KAP) related to mental health among Muslims in the LCR, and to identify actionable system leverage points to improve access, engagement and culturally responsive care.

**Methods:**

A qualitative study informed by social constructivism. Purposive sampling recruited Muslim adults (*n* = 11; age 18‐59; 6 women/5 men) from diverse backgrounds (Yemeni, Somali, Egyptian, Algerian, Pakistani, Bangladeshi). Recruitment was community‐enabled through mosques and community networks. Semi‐structured interviews (in person or MS Teams) were audio‐recorded, transcribed, anonymised and analysed using Reflexive Thematic Analysis with an audit trail, reflexive memoing and team debriefs.

**Results:**

Three interlinked themes were generated: (1) Barriers to access and engagement: stigma and reputational risk, communication difficulties, confidentiality concerns and perceived stereotyping reduced disclosure and trust; (2) Cultural and religious context in mental health: participants endorsed integrating faith‐based coping and clinical care, with mosques and imams functioning as trusted entry points but with variable mental health capability; (3) System and service provision challenges: limited knowledge of access routes, crisis visibility of services, perceived Islamophobia, and resource/leadership gaps reinforced late presentation. Findings suggested an accumulating pathway from stigma and low trust to delayed access and crisis‐driven contact.

**Conclusions:**

Inequities reflected system design and relational safety as much as individual knowledge. Co‐designed, community‐enabled pathways, faith‐literate practice, safeguarded referral interfaces with faith leaders, and routine equity monitoring are key mechanisms for improving engagement and outcomes in the LCR and similar UK city regions.

## 1 Introduction: Cultural and Religious Context in Mental Health Systems

Standard Western psychiatric frameworks can misfit ethnoculturally diverse populations when local beliefs, practices and idioms of distress do not align with diagnostic categories or service models [1, 2]. Improving equity therefore requires context‐sensitive, community‐informed care, rather than one‐size‐fits‐all approaches. A key frontier is how mental health systems engage with faith‐ and culture‐shaped meanings of distress alongside biomedical care. Qualitative evidence from Saudi Arabia shows that stigma is often intertwined with religious narratives (e.g., moral judgements about prayers/devotion and beliefs such as the evil eye), suggesting that religious literacy can be central to stigma reduction and engagement [3]. Community‐embedded models, delivering support through trusted local institutions and strengthening culturally matched provision, offer practical routes to improved access and experience [4, 5]. The Liverpool City Region (LCR) provides a strategically important setting for this work. Building on our earlier consultation [6], a Patient and Public Involvement and Engagement (PPIE) with Muslims from minoritised ethnic communities, which further identified inequities in mental health care and support and priorities for improvement [7, 8], this study uses in‐depth qualitative interviews to explore knowledge, attitudes and practices (KAP) and to generate actionable, place‐based system recommendations.

## 2 Background

### 2.1 Muslim Mental Health in Minority Contexts

Although Islam is the world's second‐largest religion, Muslim mental health remains comparatively under‐researched in English‐language literatures, particularly in minority contexts where service models and public narratives have historically been shaped by majority norms [9, 10]. Evidence from Muslim minority context, including United States, highlights heightened anti‐Muslim hostility and discrimination that can plausibly erode confidence in public institutions [11, 12]. Global evidence from conflict‐affected settings documents migration‐related stressors that increase psychological burden and shape willingness to engage with formal systems [11, 12]. This literature indicates that discrimination/Islamophobia and migration/asylum pressures may contribute to reduced trust in statutory services in minority settings. These dynamics can create ‘double stigma’ in form of community stigma about mental illness alongside wider societal prejudice related to Muslim identity [13, 14]. Despite increasing attention, much existing research is still concentrated in Muslim‐majority settings, limiting transferability to UK minority contexts [15, 16]. We therefore cite selected Muslim‐majority studies as sensitising evidence for cultural‐religious framings of stigma and distress, while treating help‐seeking pathway, system interfaces as context‐specific to UK minority settings.

In England, structural disadvantage is marked with around 40% of British Muslims living in the most deprived areas compared with 20% of the general population, with predictable impacts on mental health and access to timely care [17, 18]. The LCR is policy‐relevant, especially as Muslim communities are a minority across the region overall because (i) it combines concentrated deprivation with a growing and diverse Muslim population, and (ii) it has explicit inequalities mandate that creates a clear route for translation into practice [19, 20, 21]. The combination of need, infrastructure and system readiness makes the LCR an informative case for understanding how knowledge, attitudes and help‐seeking practices interact with service design.

### 2.2 Mental Health Literacy, Help‐Seeking and Barriers to Care

International evidence indicates Muslim mental health literacy is heterogeneous and often involves plural explanatory models (biomedical, psychosocial and spiritual/supernatural) [22, 23, 24]. In Western settings, immigrants and minority communities may hold more negative attitudes towards formal services due to stigma, perceived cultural mismatch, practical barriers and prior experiences of discrimination [25, 26]. Barriers commonly span structural factors (e.g., language, transport), social factors (stigma, racism/Islamophobia) and cultural‐religious factors (faith‐based coping preferences, moral framings of illness, beliefs such as jinn/evil eye) [27, 28, 29, 30, 31]. Within services, limited religious literacy and monocultural delivery can weaken therapeutic alliance and reduce engagement [6].

With the context of this paper, the LCR functions as a ‘living lab’ for identifying culturally responsive strategies that are feasible within a high‐need, equity‐focused system and deliverable through strong community and faith infrastructures. The aim of this study is to explore KAP related to mental health among Muslims in the LCR. Specifically, we explored attitudes to help‐seeking; identify structural, social and cultural barriers to service utilisation; describe current practices across statutory, third‐sector (voluntary organisations) and religious settings; and generate implications for culturally and religiously responsive interventions and health‐system action. UK evidence on Muslim mental health has often focused on barriers in general terms, with limited attention to how community norms, faith infrastructures and service design interact to shape engagement in a specific place. This study contributes a place‐based, systems‐oriented account from the Liverpool City Region that (i) explains how stigma, trust and service navigation accumulate to delay care; (ii) clarifies the role of mosques and faith leaders as high‐trust ‘entry points’ that can either enable or delay access depending on linkage to clinical pathways; and (iii) produces an actionable set of system leverage points (navigation support, faith‐literacy, safeguarded referral, and equity metrics) transferable to other UK city‐regions.

## 3 Methodology and Methods

### 3.1 Study Design and Approach

It is important to note that we used KAP as a pragmatic organising framework. *Knowledge* refers to participants’ understandings of mental health and of the local service system. *Attitudes* refer to evaluations and emotions shaping willingness to seek help. *Practices* refer to enacted behaviours and pathways. We recognise the overlap and therefore, report both domain‐specific findings and how domains interact. This qualitative study was therefore informed by a social constructivist position, recognising that experiences and meanings of mental health are shaped and negotiated through social, cultural and religious contexts [32]. This aligned with our aim to explore Muslim community members’ knowledge, attitudes and practices related to mental health and help‐seeking in the Liverpool City Region.

### 3.2 Recruitment

We used purposive sampling to recruit adults (≥ 18 years) who self‐identified as Muslim and were able to participate in English (See Table 1). Recruitment was supported through local mosques and community social media, including WhatsApp groups. Interested individuals contacted the first author by email or text messages, received a participant information sheet and consent form outlining the study purpose, expectations and eligibility. Written and verbal consent were obtained prior to interview.

**Table 1:** Participants demographics.

Characteristic	Category	*n*/Details
Sample size	Total	***n*** = **11**
Gender	Female	6
	Male	5
Age	Range (years)	18–59
Self‐identified background	Yemeni	4
	Pakistani	3
	Bangladeshi	1
	Algerian	1
	Egyptian	1
	Somali	1
Eligibility	Aged ≥ 18 and English‐speaking	11

### 3.3 Community Involvement (Patient and Public Involvement)

The study was community informed. Partnerships with mosques and community networks helped shape culturally appropriate recruitment and participation processes (e.g., wording of adverts, acceptable interview arrangements, safeguarding considerations). Community members were not involved as formal co‐researchers; however, interpretation was grounded in ongoing engagement with faith/community settings. An accessible summary of findings was shared with participating community organisations, and feedback was invited to inform next steps (e.g., co‐created service navigation support and principles for faith‐National Health Services (NHS) collaboration).

### 3.4 Ethical Considerations

Ethical approval was granted by Liverpool John Moores University School of Nursing and Advanced Practice Research Ethics Panel (NAH(PGT)3023; 18/04/2024). Cultural and religious sensitivities were prioritised, including managing gender dynamics where a male researcher interviewed female participants. Participants were offered alternative arrangements where preferred (e.g., remote interview), supporting autonomy and comfort [33]. We used respectful, culturally appropriate communication to build rapport and reduce barriers to participation [34]. Participants could withdraw up until analysis commenced. Distress protocols were in place and no distress was observed.

### 3.5 Data Collection: Individual Interviews

Semi‐structured interviews were conducted in person or via MS Teams at a time convenient to participants; remote interviews were completed in a private setting chosen by the participant. No third parties were present and repeat interviews were not undertaken. Interviews lasted up to 60 min and were conducted by A.T. The topic guide used open‐ended questions aligned to the study aim, was informed by a rapid review, the first and last authors’ experiential experiences of mental health nursing, community engagement, and culturally responsive practice in UK context. Interviews were audio‐recorded with verbal and written consent, transcribed, anonymised, checked for accuracy and stored securely. Recordings were deleted after verification. The guiding questions explored: (1) views of mental health support in the community; (2) help‐seeking practices; (3) perceived barriers to accessing support; (4) how services could improve access and experience; and (5) relationships between culture/beliefs, mental health and existing support.

### 3.6 Data Analysis

Sampling sought maximum variation across gender, age and ethnocultural background to capture differing help‐seeking norms and service experiences. Analysis followed reflexive thematic analysis [35]: Familiarisation with transcripts and field notes; generation of initial semantic codes; iterative clustering of codes into candidate themes; review and refinement through team discussions; final theme definition and naming of themes and reporting, with attention to convergent and divergent accounts. This audit trail (coding memos, decisions, theme iterations) supports transparency and transferability. Coding was primarily semantic, combining deductive attention to the study aims with inductive sensitivity to participants’ language. Rather than seeking inter‐coder reliability, we used reflexive dialogue and team discussion to surface assumptions and refine themes [35]. Credibility was supported through summarising and checking emergent points during interviews and inviting correction (respondent validation) [36]. Transferability and confirmability were strengthened through thick description, carefully selected quotations (including convergent and disconfirming cases) [37], and an audit trail (sampling decisions, memos, theme development notes, and team debrief summaries). Recruitment ended when further interviews were judged unlikely to add meaningful explanatory value for study aims and when the dataset offered sufficient diversity and depth to support coherent themes. We did not pursue ‘data saturation’ as in reflexive TA, saturation reflects a more positivist paradigm that fits uneasily with interpretive and iterative analysis [38].

In this manuscript, we adopt a ‘systems thinking’ approach in a bounded, qualitative sense to explore how barriers to help‐seeking arise through interactions between social norms (e.g., stigma), relational conditions (e.g., trust/communication), and service design (e.g., navigation routes, crisis visibility). We operationalised this by mapping themes onto system components and actors (community members/families, faith leaders, primary care, community mental health services, commissioners), identifying reinforcing mechanisms described in narratives and deriving leverage points that target interactions rather than isolated factors.

### 3.7 Reflexivity

The study team brought complementary expertise in academic research, mental health nursing, adult nursing, specialist public health nursing and Islamic psychology, with diverse ethnic, religious and lived experience backgrounds. A.T. (Muslim, minoritised ethnic background) and O.B. led coding, theme development and iterative refinement. Reflexivity was maintained through memo‐writing and regular critical discussions in which interpretations, assumptions and potential alternative explanations were explicitly examined [35]. I.K. provided sustained feedback from an Islamic psychology perspective, supporting accurate use of religious concepts and language and promoting wellbeing, distress and coping Islamic concepts of self and meaning. S.O., C.D.B. and A.W. stress‐tested interpretations against clinical/public health practice, safeguarding considerations and participants safety. These processes strengthen transparency, cultural sensitivity (i.e., care adapted to a person's cultural context to improve acceptability) and analytic credibility.

## 4 Results

Across themes (See Table 2), participants described an accumulating pathway that helps explain delayed engagement with statutory care. First, stigma and reputational risk constrained early disclosure, especially for men, making distress ‘private’ until it escalated. Second, when individuals considered seeking help, low confidence in being understood, due to language barriers, fear of being dismissed, and confidentiality concerns, reduced trust and increased preference for informal, high‐trust supports (family, imams, mosques). Third, experiences or anticipation of cultural/religious stereotyping signalled to participants that services may not be safe spaces to discuss faith‐framed understandings of distress. Participants’ accounts suggested an accumulating pattern in which stigma and reputational risk constrained early disclosure, while communication barriers and confidentiality anxieties reduced trust in statutory services.

**Table 2:** Themes identified.

Themes	Sub‐themes
1. Barriers to access and engagement	1.1 Stigma and normalisation; 1.2 Communication and trust; 1.3 Cultural and religious assumptions
2. Cultural and religious context in mental health	2.1 Integration of religious and medical approaches; 2.2 Religious coping and support structures; 2.3 Cultural and religious sensitivity
3. Systemic and service provision challenges	3.1 Service navigation and access points; 3.2 Over‐medicalisation vs early intervention; 3.3 Resource and leadership deficits

Table 3 links Themes 1–3 to system‐level leverage points, connecting each recommended action to the barrier mechanisms evident in participants’ narratives. These mechanisms delayed help‐seeking and made services most visible only at crisis, creating the navigation and capacity challenges outlined in Theme 3. Table 4 further shows how stigma and related barriers span knowledge, attitudes, and practices, collectively shaping help‐seeking patterns.

**Table 3:** Systems map.

System component/actor	Core issue (from current study result)	Reinforcing interaction	Leverage point (links to Table 4)
Community norms & networks	Stigma, shame, gossip risk	Concealment delays help‐seeking; late help‐seeking increases crisis contact; crisis contact reinforces stigma	Mosque/community anti‐stigma and therapy‐orientation sessions
Access routes (‘front door’)	Unclear routes; low service literacy	Unclear routes drive delay and avoidance; late entry reinforces the belief services are ‘only for crisis’	Co‐designed navigation guide (print/QR) via trusted settings
Communication infrastructure	Language barriers; fear of dismissal; confidentiality concerns	Reduced understanding and confidentiality fears reduce trust; low trust shifts help‐seeking to informal supports; informal supports delay statutory contact	Interpreter access and culturally safe communication standards
Faith infrastructures	High trust; variable mental health capability	Trust concentrates in faith settings; without pathways, capability gaps can delay clinical care	Safeguarded two‐way signposting/referral with defined roles/thresholds
Service model	Reactive, crisis‐visible support	Crisis‐driven visibility increases avoidance; avoidance increases crisis‐driven visibility	‘In‐between’ support while waiting; earlier intervention options
Commissioning/leadership	Limited sustained investment	Underinvestment sustains reactive care and limits culturally responsive provision	Equity dashboard and commissioning support for community‐enabled pathways

**Table 4:** Mapping key findings to KAP.

Key finding (from Results)	Knowledge (K)	Attitudes (A)	Practices (P)	Interaction note
Older generations less familiar with ‘mental health’ concepts	✓			Knowledge gaps sustain taboo norms and delay recognition
Mental health framed as ‘taboo’; fear of gossip/reputational harm		✓	✓	Shame/fear drives concealment and delayed help‐seeking
Low awareness of how to access NHS community mental health services	✓		✓	Limited‐service literacy shapes pathway choice and persistence
Language barriers and fear of being dismissed	✓	✓	✓	Anticipated dismissal reduces disclosure and follow‐through
Confidentiality/data anxieties about speaking to statutory services		✓	✓	Fear shifts help‐seeking toward trusted informal supports
Early reliance on prayer/mosques/imams for support			✓	Help‐seeking follows trusted, culturally congruent routes
Clinician stereotyping		✓	✓	Reduced relational safety limits disclosure and engagement

Drawing on participants’ narratives and aligned with international evidence, we identify plausible leverage points for superdiverse settings, while recognising that feasibility and accountability require local co‐design.

### 4.1 Barriers to Access and Engagement

Participants described barriers that operated before people reached statutory services and within encounters once services were accessed. These barriers were experienced as social (stigma), relational (trust and communication), and interpretive (being understood through stereotypes rather than as individuals). These experiences shaped whether distress became ‘sayable,’ who it could be said to, and whether services were viewed as safe enough to approach.

#### 4.1.1 Stigma and Normalisation

Mental health was repeatedly described as ‘taboo,’ with participants linking silence to limited education, generational norms and migration histories. One participant explained that some older generations had little exposure to mental health concepts, making recognition and conversation difficult:

Like most of the older generation is from back home […] they don't have a clue what mental health is […] how are they going to just know… without a bit of education on it?

(Participant 11, Female; Bangladesh)

Here, stigma was not presented as an abstract attitude but as a knowledge‐and‐norm system that restricts what communities can name and legitimise. This helps explain why participants later described reliance on familiar, informal supports, and why service navigation knowledge was limited (Theme 3).

Participants also characterised stigma as socially contagious, where disclosure quickly becomes community ‘talk,’ shifting distress into reputational risk:

If one person finds out […] it becomes a hot topic […] like you have something very bad.

(Participant 1, Female; Pakistan)

This implies that concealment can be a rational protective strategy, not simply denial. It also sets up the next subtheme i.e., when disclosure feels risky, people require high trust and high confidence in confidentiality, conditions that participants felt were not consistently met in statutory services. Men were described as particularly exposed to norms that equate help‐seeking with weakness, encouraging suppression until distress escalates:

It's just buried […] Men are often told to just be men and ignore it… later in life… then it becomes […] too late.

(Participant 5, Male; Egypt)

Participants suggested that stigma and disclosure norms vary within the community. Men were described as particularly constrained by expectations of strength and concerns about reputational consequences, shaping concealment and delayed help‐seeking. Participants also linked generational differences to mental health knowledge, describing older generations as less familiar with mental health concepts and services. Also, participants’ later accounts of services being most visible at crisis point (Theme 3.1) i.e., when distress is hidden early, entry into care is delayed, and the system is encountered when problems have intensified.

#### 4.1.2 Communication and Trust

Participants’ accounts suggested that trust was built or broken through everyday communication: whether symptoms were believed, whether people felt listened to, and whether confidentiality felt secure. For some, language barriers were described as producing a fear of being dismissed or ‘sent home’ without meaningful support:

If my mother tried to explain her symptoms, she'd probably get sent home saying you just need to relax […].

(Participant 11, Female; Bangladesh)

This does more than signal poor communication; it suggests that where understanding is uncertain, service contact can feel high effort and low reward, reinforcing avoidance established by stigma (1.1). It also points forward to Theme 3's navigation and access issues: if early contacts are perceived as dismissive, people may not persist through complex pathways. Interpreter access was therefore framed as a practical intervention that could restore safety and trust:

Even having an interpreter […] would just made us feel safer.

(Participant 3, Female; Yemen)

Participants treated interpreter provision as a mechanism that supports disclosure and shared understanding, conditions needed for any culturally responsive care discussed in Theme 2. Trust was also undermined by fears about data recording and the consequences of disclosure, particularly where participants perceived risks for families or within tightly connected communities. One participant described that this fear could make a faith leader feel safer than a GP:

They have got the fear that their data will be stored if they speak to the GP[…] they would feel more comfortable […] with the mullah.

(Participant 4, Female; Pakistan)

This links directly to Theme 2, where participants describe religious leaders as trusted first points of contact, and highlights why partnership and safe referral mechanisms matter, especially as people will gravitate towards relationships that feel safer, even if those relationships lack mental health expertise (Theme 2.2).

#### 4.1.3 Cultural and Religious Assumptions

Participants described feeling filtered through stereotypes and assumptions about what Muslims believe, how they understand mental health, or what their ‘culture’ supposedly dictates. This was experienced as diminishing credibility and weakening therapeutic connection. Majority of participants emphasised that high respect for doctors can intensify the impact when mental health needs are not met with nuance:

Muslims look up to doctors […] but when it comes to mental health […] people will rely a lot on the doctors.

(Participant 11, Female; Bangladesh)

This suggests a sharp contrast between expectation and experience: when trust is culturally granted to professional authority, perceived misunderstanding can lead to rapid disillusionment, feeding back into avoidance and reliance on informal pathways. Some participants gave direct examples of micro‐assumptions that felt dismissive:

She wears a headscarf; she probably doesn't really understand.

(Participant 4, Female; Pakistan)

Others described being asked to confirm stereotypes (e.g., that Muslims attribute distress to ‘devils’), which made them feel unheard:

Not every Muslim believes that mental health is caused from devils […] that puts you down […] because you think no one's really listening.

(Participant 11, Female; Bangladesh)

These accounts set up Theme 2 as they show why cultural and religious sensitivity is not simply about ‘being nice,’ but about whether services can hold faith‐related narratives respectfully enough for people to disclose. When assumptions close down conversation, participants’ preference for faith and community support becomes more understandable.

### 4.2 Cultural and Religious Context in Mental Health

Participants described religion as shaping meaning, coping and where people go first for support. Rather than positioning faith as competing with clinical care, many emphasised the value of a combined approach, where spiritual practices support hope and meaning while clinical care addresses symptoms and functioning. This theme also revealed a tension i.e., faith leaders are trusted and accessible but may not always be equipped to respond safely, linking back to Theme 1's trust dynamics and forward to Theme 3's system design and resourcing issues.

#### 4.2.1 Integration of Religious and Medical Approaches

Participants commonly described faith and medicine as complementary. One participant framed this as something services should recognise and enable:

So, religion is important to me […] If medication is important to you, then you can combine them as well.

(Participant 11, Female; Bangladesh)

The implication here is that culturally responsive care is not about replacing evidence‐based treatment, but about designing services so that faith‐consistent coping and clinical approaches can coexist without judgement. This also speaks back to Theme 1.3: when clinicians assume faith explanations are irrational or ‘wrong,’ they may inadvertently block engagement. Several participants proposed community‐based conversations in mosques as practical routes to reduce stigma and improve awareness of where to seek help:

Local mosques […] bring more people to talk about mental health […] remind people […] where they can go to when they need help.

(Participant 3, Female; Yemen)

This anticipates Theme 3.1: low awareness of access routes was a major barrier, and participants positioned mosques as credible venues for navigation support and normalisation.

#### 4.2.2 Religious Coping and Support Structures

For some participants, prayer was described as immediate, accessible comfort:

If I have a problem, the first thing I do is pray and I feel better.

(Participant 9, Male; Somalia)

This illustrates why people may begin within faith frameworks before approaching statutory services. However, participants described tension between trust and expertise in relation to Imams and community leaders. One participant cautioned that visible markers and positional status can confer unexamined authority:

Anybody who has a beard […] wears a long thobe […] is seen […] as […] authority.

(Participant 5, Male; Egypt)

They also highlighted that leaders may lack formal mental health training:

Leaders […] are extremely unqualified […] [with] no formal training in mental health.

(Participant 5, Male; Egypt)

These accounts suggest that faith leaders can function as trusted first contacts and opinion‐shapers, but without supported pathways they may inadvertently delay evidence‐based care. This links forward to Theme 3.3: participants later emphasised the need for resourcing, leadership and commissioning support to create safe, collaborative interfaces rather than leaving communities to manage risk alone. Participants also argued that culturally appropriate conversations with professionals could improve approachability, including training that helps staff discuss mental health in ways that resonate with faith‐framed understandings:

Maybe the staff could get training on what Islam is all about… that within itself will make you feel comfort if religion is important to you.

(Participant 11, Female; Bangladesh)

This connects back to Theme 1.3 (assumptions) and indicates a pathway for rebuilding trust.

#### 4.2.3 Cultural and Religious Sensitivity

Participants described cultural sensitivity as foundational to engagement. They questioned whether services recognised the need to ‘draw people in’ through education and relationship‐building, or whether staff operated from stereotypes:

Do they realise that they might need that education to draw them in to the service? […]. Have they got their own assumptions […] they think Muslim people think mental health is caused from the devil […].

(Participant 11, Female; Bangladesh)

This implies that sensitivity is about accommodating practices and how services interpret service users and communicate respect. Without this, the trust and communication problems described in Theme 1 are likely to persist, increasing the probability that services are encountered late and in crisis (Theme 3).

### 4.3 Systemic and Service Provision Challenges

Participants described a mental health system that felt difficult to enter, reactive rather than preventive, and uneven in cultural responsiveness. System issues, unclear access routes, perceptions of Islamophobia, and limited investment in community‐based early support, appeared to amplify the social and relational barriers described earlier, reinforcing a cycle of delayed help‐seeking and crisis‐based contact.

#### 4.3.1 Service Navigation and Access Points

Participants reported limited knowledge of how to access community mental health services and described statutory mental health care as most visible at crisis point:

The only time you see mental services is at crisis point… with Merseyside police.

(Participant 9, Male; Somalia)

This supports the pattern implied across Themes 1 and 2: when stigma discourages early disclosure and trust is uncertain, entry into care may occur only when symptoms escalate. Concerns about Islamophobia and stereotyping further reduced willingness to engage early. Participant described a racist ‘joke’ by a professional and how that shaped fear about being allocated to a biased clinician:

What if they're biased? What if they're racist towards me?

(Participant 8, Female; Yemen)

Another noted how negative media portrayals fuel anticipatory judgement and identity management (e.g., shaving beards to avoid suspicion):

Cousins […] have shaved it off because they think that they're being judged.

(Participant 11, Female; Bangladesh)

These accounts highlight how wider social climates enter clinical spaces, shaping trust and engagement well before appointments occur.

#### 4.3.2 Over‐Medicalisation Vs Early Intervention

Participants described a perception that care is overly medication‐focused, sometimes reinforced by prior experiences in settings where healthcare is paid for and prescriptions are expected as ‘proof’ of care:

I have paid… and I was not given any prescription; this doctor is no good.

(Participant 4, Female; Pakistan)

This does not imply rejection of medication; rather, participants advocated for a more holistic and preventive offer (e.g., social prescribing and early support), and for services that can reach communities sooner. Participant emphasised the need for well‐led, well‐funded teams that work at grassroots level to prevent escalation:

We need a good team […] access to all the communities […] catch people early.

(Participant 9, Male; Somalia)

This links back to Theme 2.1's emphasis on community settings and to Theme 1's finding that stigma drives delay: without early intervention options, the system itself becomes crisis‐oriented.

#### 4.3.3 Resource and Leadership Deficits

Participants attributed gaps in culturally responsive community provision to insufficient funding, weak commissioning support and limited leadership engagement with Muslim communities. One participant described previous attempts to create interim support for people waiting for care, which failed due to lack of commissioner backing:

We need a system in the middle… but that itself needs a lot of money… didn't work because there was no Commissioners to support.

(Participant 9, Male; Somalia)

This speaks to sustainability as participants’ proposed solutions (e.g., mosque‐based outreach, faith‐literate training, navigators, early intervention) require not only goodwill but structural investment and leadership. Without this, communities remain reliant on informal support and episodic crisis responses, reinforcing the barriers described in Theme 1

## 5 Discussion

This study explored mental health knowledge, attitudes and practices among Muslims in the Liverpool City Region (LCR), a population underrepresented in UK mental health research. Participants from Yemeni, Somali, Egyptian, Algerian, Pakistani and Bangladeshi backgrounds described intersecting barriers to help‐seeking and engagement, shaped by stigma, trust and communication, and the ways services interpret culture and religion. Our findings indicate that inequities are driven by individual ‘health literacy’, and by whether mental health systems invite, recognise and respond to help‐seeking in culturally safe and faith‐literate ways.

### 5.1 Contribution to Knowledge and Practice

This study makes three contributions. Conceptually, it advances a systems account of Muslim mental health engagement in a UK city‐region by showing how stigma, trust/communication and cultural assumptions interlock with unclear access routes to produce late, crisis‐driven contact. Methodologically, it demonstrates a community‐informed qualitative approach that treats faith settings not as ‘alternatives’ to care but as engagement infrastructures that shape candidacy for services. Practically, it specifies implementable leverage points: (1) mosque/community‐delivered service‐navigation guides and therapy orientation; (2) faith‐literacy training plus reflective supervision prompts for clinicians; (3) safeguarded, two‐way referral/signposting protocols with clearly defined roles and thresholds; and (4) equity tracking access, experience and outcomes by ethnicity/language (and faith where appropriate and acceptable). These contributions are transferable to other deprived, superdiverse UK contexts seeking to reduce unequal engagement and improve culturally responsive provision.

### 5.2 Plural Explanatory Models and Help‐Seeking Pathways

Prior work shows that many Muslims draw on plural explanatory models that combine biomedical, psychosocial and spiritual understandings of distress [22, 23]. In this current study, this pluralism shaped practices in that people often began with prayer and trusted community support, approaching statutory care later when problems escalated. This perhaps helps explain why service navigation knowledge remained limited and why crisis visibility became a dominant ‘entry point’ experience.

### 5.3 Stigma, Trust and Cultural Assumptions

Participants highlighted multi‐level stigma (self, family and community), fears about reputational harm, and moral framings that associate mental illness with weak faith or personal failure [39, 40, 41]. These concerns were compounded by low trust in services and perceived dismissal, particularly where language barriers and confidentiality anxieties limited disclosure. Structural barriers, transport, time, costs, childcare and immigration‐related insecurity, are well documented [22, 27, 28], and are likely amplified in a high‐deprivation context such as the LCR [17, 18]. In this current study, stigma was described as a reputational risk that encouraged concealment and delayed help‐seeking, particularly for men. Participants also described frustration when clinicians assumed faith‐based explanations (e.g., ‘devils’) rather than inviting respectful discussion, which reduced relational safety and disclosure. Importantly, religiosity was not inherently a barrier as participants positioned faith as a coping resource and a route to trusted support, reinforcing the need for faith‐literate encounters (ability to discuss faith respectfully) [42]. Building trust therefore requires an explicit shift from monocultural delivery to culturally responsive, relationship‐based practice.

### 5.4 Faith Coping and Engagement Infrastructure

Participants positioned faith leaders as high‐trust actors who can reduce stigma and enable early conversations but also highlighted risks where religious authority is assumed to equal mental health expertise. We therefore conceptualise mosques and faith leaders as engagement infrastructure rather than treatment providers. In other words, they can facilitate safe disclosure, community education, and supported signposting, but should not carry clinical responsibility for diagnosis, risk assessment, safeguarding, or treatment decisions. Any interface should include explicit boundaries (what faith leaders can/cannot do), defined referral thresholds, escalation routes, mutual safeguarding agreements, and clear accountability within statutory services. Our framing recognises the social capital of faith settings without romanticising them and addresses power dynamics by anchoring responsibility for safety and care planning within NHS systems. Informed by participants’ accounts in this current study and supported by relevant policy and implementation literature, we propose the following as plausible leverage points to improve engagement and equity. These recommendations extend beyond the dataset and should be tested through co‐design with community members, clinicians, commissioners and faith leaders.

### 5.5 Implications for Implementation and Equity

Drawing on a knowledge‐to‐action perspective [43], the findings point to practical steps for improving engagement and outcomes through co‐designed, community‐enabled approaches. Cultural responsiveness should not depend on individual practitioners; it requires system‐level design across commissioning, workforce development, supervision and performance monitoring [44]. Table 5 translates Themes 1‐3 into system‐level leverage points by linking each recommended action to the barrier mechanism evidenced in participant narratives.

**Table 5:** Plausible leverage points to improve engagement and equity for Muslim communities.

Barrier/pinch point	System mechanism	Engagement‐focused action	Lead actor(s)
Unclear access routes	Navigation friction; limited‐service literacy	Co‐design a simple ‘how to access help’ guide delivered via mosques/community groups (print + QR).	Providers; commissioners; community partners
Low trust/fear of judgement	Relational safety deficit; prior negative experiences	Community‐hosted listening sessions and ‘you said ‐ we did’ feedback loops into services.	Providers; VCSE; faith/community leaders
Limited faith literacy in services	Capability gap; inconsistent practice	Faith‐sensitive training, supervision prompts and reflective practice tools embedded in teams.	Providers; educators; professional leads
Over‐reliance on informal support	Parallel pathways; unclear referral boundaries	Safeguarded two‐way signposting/referral protocols with defined roles, thresholds, documentation and escalation routes; agreed safeguarding principles and training	Providers; safeguarding leads; faith leaders
Stigma and moral framing	Social norms; reputational risk	Co‐produced anti‐stigma messaging aligned with faith values; peer champions; opt‐in family‐inclusive sessions.	Public health; community organisations; faith leaders
‘Invisibility’ in metrics	Accountability gap	Routine equity monitoring (ethnicity/language/faith where appropriate); qualitative feedback on experience/trust.	Commissioners; providers

Across these actions, nurses and allied health professionals are well placed to lead the relational interface – coordinating care, supporting culturally safe communication, and maintaining risk management, confidentiality and evidence‐based treatment.

### 5.6 Strengths, Limitations and Future Directions

A key strength is the analytic coherence achieved through reflexive thematic analysis in a social constructivist tradition, supported by an audit trail and team reflexive dialogue. This enabled the ‘accumulating pathway’ to be presented as an interpretive heuristic grounded in participant narratives to guide subsequent co‐design and feasibility testing. Three limitations warrant emphasis. First, Muslims in the LCR are not a homogeneous group; ethnicity, language, migration status, sect, age and gender shape KAP in distinct ways. Future studies should stratify analyses to avoid over‐generalisation and to identify which engagement strategies work for whom. Second, place‐based findings are time‐sensitive: trust and perceived safety can shift with media cycles and policy change. Iterative community engagement and evaluation are therefore essential. Third, this study foregrounds community perspectives. We did not include interviews with clinicians, commissioners, or faith leaders. As such, feasibility, accountability and implementation constraints of proposed leverage points (e.g., safeguarded referral interfaces, equity dashboards) require further co‐design and testing with system stakeholders. Future work should incorporate multi‐stakeholder perspectives and rapid‐cycle evaluation to assess acceptability, safeguarding, and operational delivery.

Where refugee/asylum literature is referenced, it is used to indicate how legal insecurity, and displacement can intensify barriers for some Muslim subgroups; we do not assume these conditions apply to all Muslims in the LCR. Eligibility for current study required English fluency, therefore, accounts of language barriers primarily reflect participants lived‐experience, observations of family/community experiences and anticipated challenges, rather than direct experiences of non‐English‐speaking participants. Future work should include multilingual recruitment and interpreter‐supported interviews to capture barriers experienced directly by those most affected. Although our dataset did not support formal subgroup comparison, participants’ accounts indicate that gender and age may shape both the intensity and form of stigma and should be examined explicitly in larger, stratified studies. Proposed studies will likely require broader recruitment beyond mosque networks and multilingual methods to reach less‐connected subgroups, including those most affected by language barriers. While the LCR context is distinctive, alignment with international evidence already cited suggests potential relevance to other deprived, superdiverse settings; nonetheless, transferability should be assessed through local stakeholder co‐design, safeguarding review and feasibility evaluation.

## 6 Conclusion

This study suggests that inequities reflect system design and relational safety as much as individual knowledge. The leverage points identified may be relevant to other deprived, superdiverse UK city‐regions, but require local co‐design and feasibility. Community participation and co‐designed service interfaces, mobilising trusted settings such as mosques while maintaining safeguarding and clinical oversight are central to improving equity in access, experience and outcomes. Services should invest in faith‐literate, culturally responsive care; reduce practical barriers to access; integrate plural care pathways through clear collaboration protocols/standard operating procedures; and make equity visible through routine monitoring and improvement cycles.

## Author Contributions

**Ashraf Tannerah:** conceptualisation, methodology, validation, investigation, funding acquisition, writing – original draft, project administration, data curation, formal analysis. **Amy Webster:** validation, investigation, manuscript review. **Shelley O'Connor:** validation, investigation, manuscript review. **Charlie Douglas‐Brown:** validation, investigation, manuscript review. **Imran Khan:** validation, investigation, manuscript review. **Oladayo Bifarin:** investigation, conceptualisation, methodology, validation, visualisation, writing – review and editing, formal analysis, supervision, data curation, resources.

## Conflicts of Interest

Oladayo Bifarin is a National Institute for Health and Care Research Leader. The views expressed in this article are those of the author(s) and not necessarily those of NIHR or the Department of Health and Social Care.

## Data Availability

The data that support the findings of this study are available from the corresponding author upon reasonable request.
